# Updates on Imaging of Common Urogenital Neoplasms

**DOI:** 10.3390/cancers17010084

**Published:** 2024-12-30

**Authors:** Athina C. Tsili

**Affiliations:** Department of Clinical Radiology, Faculty of Medicine, School of Health Sciences, University of Ioannina, 45110 Ioannina, Greece; atsili@uoi.gr

Urogenital neoplasms represent some of the most common malignancies. Advances in imaging have given radiologists an increasingly significant role in the diagnosis, staging, treatment planning, and follow-up of patients with urogenital tumors.

In this Special Issue, the value of current imaging techniques is emphasized, including ultrasonography (US), computed tomography (CT), magnetic resonance imaging (MRI), and fluorodeoxyglucose (FDG)–positron emission tomography (PET)–CT, and the potential applications of novel imaging tools in the work-up of patients with urogenital neoplasms are discussed. This Special Issue contains four original studies and six reviews, which I briefly present in the following paragraphs.

Endometrial cancer is the most common gynecologic neoplasm in the United States and Europe, with incidence rates increasing by about 1–3% per year [1,2]. The updated 2023 FIGO staging system incorporates histological and molecular classifications to better reflect the complex nature and biological behavior of the different types of endometrial cancer, providing a more evidence-based context for treatment planning [3]. Although endometrial cancer is a surgically staged disease, preoperative MRI is recommended as it provides critical diagnostic information on tumor size and depth, the extent of myometrial and cervical invasion, extrauterine extent, and lymph node status, all of which are important in the planning of an appropriate treatment [1,2,4,5].

The important role of preoperative MRI in the local staging of endometrial cancer is validated in the original study of Van Vynckt et al. (contribution 2). The authors found that MRI has a good diagnostic performance for the detection of ≥pT1b endometrial cancer (i.e., tumor with an invasion of half or more of the myometrium, invasion of the cervical stroma, or extrauterine spread). The ≥pT1b threshold is one of the most important clinical factors in the T-staging of endometrial carcinoma, as it has significant prognostic value and direct implications for planning lymphadenectomy in addition to hysterectomy. In the same study, tumor size was proven to be a predictive factor of ≥pT1b disease, irrespective of MRI signs of invasion. Specifically, a tumor diameter measured via an MRI of ≥40 mm and a tumor volume of ≥20 mL proved highly predictive for the presence of ≥pT1b disease. More importantly, an endometrial carcinoma size of at least 5 mm was associated with ≥pT1b disease in more than 50% of cases, confirming the value of the size criterion as an independent prognostic factor of endometrial carcinoma and as a guide to determine the most appropriate surgical strategy.

Cervical cancer is the fourth most common carcinoma in women worldwide [6,7,8]. Despite advances in prevention and treatment, morbidity, and mortality in women with cervical cancer remain high. MRI is the preferred imaging modality in staging cervical cancer and is included into the 2018 FIGO staging system. The primary aim of the FIGO system is to risk stratify patients who are eligible for primary surgery and those who will benefit from chemoradiation. MRI allows for the accurate assessment of tumor size, parametrial and vaginal invasion, lymph node involvement, and urinary bladder and bowel invasion [6,7,8].

Shakur et al. (contribution 8) highlight MRI findings and pitfalls corresponding to the 2018 FIGO classification of cervical cancer and discuss their implications on treatment selection. This review also comments on the efficacy of MRI in assessing which patients are eligible for fertility-sparing surgery. For women with cervical cancer treated with chemoradiation, the authors present the role of MRI in radiotherapy planning alongside image-guided adaptive brachytherapy, the assessment of treatment response, the detection of tumor recurrence, and treatment complications.

Characterization of an ovarian mass is an important part of pretreatment evaluation. The referral of women with ovarian cancer to a gynecologic oncologist significantly improves survival rate. However, in cases of benign ovarian masses, alternative treatments, such as laparoscopic surgery or surveillance, may be recommended [9,10,11]. MRI is a highly accurate technique in the detection, localization, and characterization of ovarian masses, mainly used in cases of sonographically indeterminate adnexal mass lesions, allowing for appropriate subspecialty referral and optimal preoperative planning [9,10,11,12,13]. The introduction of the Ovarian-Adnexal Reporting and Data System (O-RADS) for MRI was a significant advancement in the work-up of patients with ovarian masses, standardizing the reporting of ovarian lesions, increasing diagnostic accuracy, and helping in the stratification of malignancy risk [12,13]. In the narrative review by Bourgioti et al. (contribution 9), discriminative MRI features of common and uncommon ovarian and non-ovarian pelvic masses are presented, providing helpful tips for lesion characterization. The authors also describe a stepwise approach to lesion localization, as the first question faced by radiologists when evaluating a suspicious pelvic mass is to suggest its origin, whether ovarian or extraovarian. In the same review, a special emphasis is placed on MRI features of ovarian masses detected in the pediatric population and during pregnancy [14,15,16,17,18,19].

Panico et al. (contribution 3), in their retrospective study, assessed the diagnostic accuracy of unenhanced MRI in the characterization of ovarian masses with inconclusive US findings in pregnant women. The study used both subjective assessment and a Non-Contrast MRI Score (NCMS) assessed by two radiologists with different expertise in gynecologic imaging. The NCMS is a quantitative tool, introduced in the literature by the same authors for the characterization of adnexal masses detected during pregnancy [20]. Although most adnexal masses seen in pregnancy are benign, malignant tumors are detected in approximately 1–8% of cases [17,18,19]. MRI represents the preferred imaging modality for the characterization of adnexal tumors in pregnant women, when sonographic findings are indeterminate [17,18,19]. The use of intravenous gadolinium-based contrast agents in pregnancy should be restricted to cases in which the potential benefits of MRI significantly outweigh the potential risks to the fetus [21,22]. This renders unenhanced MRI an important tool for the characterization of ovarian masses in this population. In the study, NCMS was proven to be a reliable tool in predicting the risk of malignancy in adnexal masses during pregnancy. More importantly, this score was extremely helpful for inexperienced radiologists, and therefore, could be used in centers not specialized in gynecologic imaging.

Peritoneal metastases represent the most common pathway for the spread of primary or recurrent ovarian cancer. Diagnostic work-up with CT, MRI, and/or FDG PET/CT plays a vital role in the accurate evaluation of the extent of peritoneal carcinomatosis in women with ovarian cancer. The accurate mapping of peritoneal metastases is pivotal in planning the appropriate therapeutic strategy, predicting the likelihood of optimal cytoreduction, and identifying potentially unresectable or difficult disease sites that may require surgical technique modifications [23,24].

Based on recently published joint recommendations by the European Society of Gastrointestinal and Abdominal Radiology, the European Society of Urogenital Radiology, the Peritoneal Surface Oncology Group International, and the European Association of Nuclear Medicine, MRI is considered the most accurate imaging technique to assess the extent of peritoneal metastases in ovarian cancer [23]. MRI allows for a better detection of subcentimeter peritoneal metastases and peritoneal carcinomatosis, involving certain anatomic areas, such as the bowel serosal surface, pelvis, right hypochondrium, and mesentery [24,25,26,27]. CT has limitations in the assessment of peritoneal carcinomatosis, including poor soft tissue contrast and diminished sensitivity in the detection of small peritoneal metastases and those in certain anatomic locations, including the mesentery and bowel serosa, especially in the absence of ascites [24,25,27]. However, CT is often used for the initial staging of ovarian cancer, treatment response monitoring, and evaluation of the extent of the disease in suspected recurrence, mainly due to its widespread availability [23]. The disadvantages of FDG PET/CT in the evaluation of peritoneal metastases include limited spatial resolution in the detection of small implants; difficulty in the evaluation of diffuse peritoneal disease; the presence of neoplasms with low FDG avidity, such as mucinous tumors; false positives due to inflammation, infection, and/or the normal physiologic activity in the bowel, gallbladder, vessels, ureters, and urinary bladder; limited availability; and high cost [25,27,28]. Therefore, FDG PET/CT is often used as a problem-solving technique in women with ovarian cancer suspected of recurrence, as it can help to detect extraperitoneal metastases that were potentially missed on prior imaging [23].

The superiority of MRI in the detection of peritoneal metastases in ovarian cancer is reported in an up-to-date systematic review and meta-analysis (contribution 10), comparing the diagnostic performance of multidetector CT, MRI (including diffusion-weighted imaging), and FDG PET/CT. Based on the results of this meta-analysis, MRI and FDG PET/CT had higher diagnostic performances in the detection of peritoneal metastases compared to CT on a per patient analysis. On a per lesion basis, sensitivity estimates were similar for all imaging modalities.

Based on a thorough literature search, Miceli et al. (contribution 6) review the diagnostic performance of traditional imaging modalities, including CT, MRI, and PET/CT in the detection of peritoneal carcinomatosis in patients with advanced ovarian cancer. The authors also present classification systems useful in diagnostic evaluation—including the Peritoneal Cancer Index and the Bowel, Upper Abdomen, Mesentery in Peritoneal Metastasis score—and describe diffusion pathways, the most frequent patterns of disease, and anatomic sites that are difficult to evaluate on imaging [24,29]. Comments on evolving imaging tools in the assessment of peritoneal metastases in ovarian cancer are included, such as PET/MRI and radiomics.

Although most intratesticular masses should be considered malignant, a possible diagnosis of benign testicular lesions substantially improves patient care and may decrease the number of unnecessary radical surgical explorations. Conventional US, including grayscale and color Doppler US, represents the imaging modality of choice for the assessment of testicular masses, with high diagnostic accuracy in lesion detection and characterization. However, US does not always allow for a confident characterization of the nature of an intratesticular mass [30,31].

Multiparametric US, including conventional grayscale and color Doppler US, contrast-enhanced US, and elastography, introduced into clinical practice in the last two decades, has greatly improved the diagnostic efficacy of US in the assessment of testicular diseases [32,33,34]. Pozza et al. (contribution 7) present a detailed roadmap of the multiparametric US features of several common and uncommon benign and malignant testicular lesions that is useful in daily clinical practice. This pictorial review, based on an extensive Medline search, describes the clinical features, conventional US, contrast-enhanced US, and elastography findings of intratesticular masses that are helpful for lesion characterization.

Huang et al. (contribution 1) confirmed the adjunct role of multiparametric US in the increase in the diagnostic confidence in the characterization of focal intratesticular lesions by incorporating the 10-year experience of a tertiary center. The study includes the largest cohort published up to date.

The absence of contrast enhancement is considered as one of the most sensitive signs for predicting the benign nature of intratesticular masses. Color Doppler US may not depict blood flow in a testicular tumor, especially those with a diameter less than 1.5 cm [32]. Contrast-enhanced US can more reliably distinguish between vascularized and avascular focal testicular lesions, and therefore helping to exclude malignancy [32]. Dean Huang’s study reported the presence of vascularity in all malignant testicular tumors on contrast-enhanced US, including cases of malignancies that were detected as “avascular” on color Doppler US. In addition, by using time-intensity curves, the authors showed the efficacy of contrast-enhanced US in the characterization of intratesticular tumors, and particularly in the differentiation between seminomas and benign Leydig cell tumors.

The widespread use of scrotal sonography in recent years has resulted in a rise in the detection of small, impalpable, incidentally found testicular tumors [33,35]. These lesions are often benign, and Leydig cell tumors with low malignant potential represent the most common histologic type. The preoperative characterization of Leydig cell tumors based on imaging findings is important, as conservative treatment may be strongly recommended. The authors found a distinct vascular pattern of prolonged enhancement on contrast-enhanced US, which is highly suggestive of the diagnosis of Leydig cell tumors.

Elastography is recognized as an essential part of the multiparametric US of the scrotum, providing additional information on tissue stiffness, aiming to further improve diagnostic efficacy in the characterization of testicular lesions [34]. Although a significant overlap exists between the elastographic characteristics of testicular mass lesions, Huang showed that the combined use of contrast-enhanced US and elastography had a higher specificity in the differentiation between malignant and benign testicular abnormalities compared to conventional US.

The increased use of cross-sectional imaging over the last few decades has resulted in a rise in the number of incidentally detected renal tumors and an increase in the incidence of renal cell carcinoma. Imaging has a pivotal role in the detection and characterization of renal tumors, as well as in the staging, prognosis, therapeutic management, and follow-up of patients with renal cell carcinoma [36,37].

Innovative imaging techniques have been introduced into clinical practice, aiming to improve the efficacy of conventional imaging in the work-up of renal masses [36,37,38]. The review by Bellin MF et al. (contribution 5) discusses the most promising, novel imaging approaches in renal cell carcinoma diagnosis, including Dual-Energy CT; Photon-Counting Detector CT; multiparametric MRI; contrast-enhanced US; innovative nuclear medicine techniques, such as sestamibi SPECT/CT and PMSA PET/CT; radiomics; and Artificial Intelligence. The authors also comment on recently proposed or updated imaging algorithms and guidelines used for the diagnosis of renal cell carcinoma, including the Bosniak Classification of Cystic Masses, Version 2019; Clear Cell Likehood Score for the characterization of solid renal tumors, based on multiparametric MRI findings; and the 2017 American Urological Association recommendations, focused on the evaluation and management of clinically localized sporadic renal masses suspicious for renal cell carcinoma in adults and renal mass biopsy.

Sarcopenia, the progressive, generalized skeletal muscle disorder characterized by a reduction in muscle mass and strength, develops as a consequence of the progression of cancer cachexia in oncologic patients. Sarcopenia may be used as an important biomarker in the work-up of patients with urogenital tumors [39,40,41]. The study by Borrelli et al. (contribution 4) describes an easy-to-use CT-based Artificial Intelligence-powered software assessing sarcopenia in patients with advanced urothelial neoplasms that may be used as a reliable predictor of clinical benefits in terms of tumor response to systemic chemotherapy and oncologic outcomes.

Sarcopenia is often measured by a cross-sectional skeletal muscle area in a single CT slice, more often at the level of the third lumbar vertebra. However, this technique is time-consuming and the measured single-slice area provides only an estimation of the total muscle mass [42]. CT-based Artificial Intelligence models can automate body composition and sarcopenia measurement, helping clinicians to offer a more tailored treatment to patients [43,44,45].

Artificial Intelligence and radiomics is an emerging field of research that aims to offer significant advancements in the diagnosis, prognosis, and management of urogenital malignancies [46,47,48,49,50,51,52,53]. Radiomics allows for a high throughput extraction of quantitative data from images, capturing the complex tissue microstructure; improving detection and characterization of malignancies, the determination of tumor grades, and metastatic potential; and predicting survival rates and risks of recurrence. Radiomics analysis and its integration with clinical data and other quantitative biologic information, such as genomics and proteomics, are expected to enhance both precision and personalization in medical treatments [46,47,48,49,50,51,52,53].
